# Silver–Graphene Oxide Nanohybrids for Highly Sensitive, Stable SERS Platforms

**DOI:** 10.3389/fchem.2021.665205

**Published:** 2021-06-07

**Authors:** Mateusz Kasztelan, Anna Studzinska, Grażyna Zofia Żukowska, Barbara Pałys

**Affiliations:** ^1^ Faculty of Chemistry, University of Warsaw, Warsaw, Poland; ^2^ Chemical Faculty, Warsaw University of Technology, Warsaw, Poland

**Keywords:** plasmon, Raman spectra, ammonia solution, basic solution, infrared, poly-o-aminothiophenol, surface enhanced Raman spectroscopy), noble metal nanoparticles

## Abstract

Graphene oxide–silver nanoparticle nanohybrids were synthesized by simple reduction of the silver nitrate and graphene oxide (GO) mixture in water using the mild reducing agent ascorbic acid. The concentration of ascorbic acid was varied to verify the possible influence of the GO surface composition on the efficiency of the hybrid material as substrates for surface enhanced Raman spectroscopy (SERS). Furthermore, the composites were conditioned in ammonia solution or in potassium hydroxide diluted solution. For comparison, the graphene oxide–silver nanoparticle composite has been synthesized using the ammonia-treated GO. All materials were characterized using spectroscopic and microscopic methods including UV–Vis, infrared, and Raman spectroscopy and scanning electron microscopy. The SERS efficiency of the nanohybrids was tested using 4-aminothiophenol (PATP). The optimal synthesis conditions were found. Ammonia and potassium peroxide drop-casted on the composite changed the SERS properties. The sample treated with KOH showed the best SERS enhancement. The variation of the SERS enhancement was correlated with the shape of the UV–Vis characteristics and the surface structure of the composites.

## Introduction

Surface enhanced Raman spectroscopy (SERS) shows exceptional sensitivity, enabling detection of analytes at micromolar or lower concentrations (Zhang et al., 2017). Under optimized conditions, even single molecules can be studied (Zrimsek et al., 2017). Thanks to the fingerprint specificity of Raman spectra, SERS is successfully used in detection of viruses (Luo et al., 2014; Maddali et al., 2021), bacteria (Lin et al., 2014; Dina et al., 2021), and toxins in biological and environmental samples (Li et al., 2016). SERS probes designed with antigens or aptamers are used for the highly sensitive detection of cancer cells and biologically important molecules (Wu et al., 2012; Darrigues et al., 2017).

Such a wide field of applications of SERS stimulates research on new SERS-active materials to ensure the selectivity, sensitivity, and repeatability of SERS probes. Extensive efforts are directed to find all factors influencing the enhancement mechanism and the stability of SERS-active materials. Until now, it was generally agreed that localized surface plasmon resonance is responsible for the largest part of the enhancement of the Raman signal (Langer et al., 2020). Another factor is the chemical effect resulting from the charge transfer between the SERS-active material and the adsorbed molecule. The chemical enhancement is much lower than the plasmon resonance contribution, but it attracts significant attention because it operates not only on metals but also on semiconductors and carbon nanomaterials (Otto, 2005; Lombardi, 2017). Aside from the two main mechanisms, the dipole contribution from polar surface groups is also taken into account. The dipole moments induced by the electromagnetic radiation enhance the electromagnetic field in the vicinity of the SERS-active surface (Wang et al., 2020).

Silver nanostructures belong to the most effective enhancers of the Raman signal, although the very high enhancement factors are compromised by limited stability (Berbeć et al., 2019). A possible way to achieve highly efficient and stable SERS supports is employing silver and silver oxide hybrid nanostructures (Zou et al., 2019). Combining silver nanoparticles with graphene family materials is another way to improve the stability and repeatability of SERS enhancement (Mohammadi et al., 2018). Graphene itself enhances the Raman intensity of adsorbed molecules (Zhang et al., 2016) and dumps the fluorescence background significantly, which often obscures the Raman spectra. Another member of the graphene family—graphene oxide (GO)—also has the ability of fluorescence quenching. It also has the ability of the enhancement of the Raman signal by the chemical mechanism, similar to graphene. The unique feature of GO is the presence of the polar oxygen groups on its surface. The polar groups contribute to the SERS activity, generating the local dipole field upon interaction with the laser beam (Wang et al., 2020). The presence of the polar groups also influences the adsorption of studied molecules. GO can be gradually reduced by chemical or electrochemical methods, providing the unique ability to tune the physicochemical properties of the surface.

Despite the extensive research published on the hybrid materials based on noble metal nanoparticles and graphene, the physicochemical properties of these materials are not fully elucidated yet (Kavitha, 2018). The SERS enhancement and other optical properties of the composites depend on many factors. The shape of silver nanostructures influences their plasmonic properties and, consequentially, the SERS enhancement factors. Among silver nanospheres, nanocubes, and nanoctahedra combined with GO, the octahedral nanoparticles showed the highest enhancement ability (Fan et al., 2014). Dai et al. (2015) have shown that the number of graphene layers covering the silver bowtie nano-antenna arrays influences the photocatalytic conversion of para-aminothiophenol (PATP) into p,p’-dimercaptoazobenzene, suggesting that the number of graphene layers has an important contribution to the optical characteristics of the hybrid materials. Yang et al. (2013) compared the SERS properties of graphene, GO, and reduced graphene oxide (rGO). It has been shown that the enhancement of the Raman signal increases with the increasing number of GO layers, but an opposite rule is valid for graphene or rGO, indicating that oxygen surface groups, present in GO, are important for the SERS properties. Typically, GO shows higher enhancement than rGO (Yu et al., 2011; Yan et al., 2017; Wang et al., 2018), but there are exceptions to this rule. Mohammadi et al. (2018) have shown that in the case of composites of rGO and silver nanodendrites, the reduction of GO improves the SERS signal.

Li et al. (2015) synthesized the silver nanoparticle (AgNP)—graphene oxide hybrid by the reduction of a mixture of AgNO_3_ and GO with sodium citrate. The hybrid film composed of reduced GO (rGO) and AgNP hybrid film was then fabricated by evaporating the reaction mixture solution and harvesting the film formed at the air–liquid interface with a solid substrate. The SERS performance of such nanohybrids was dependent on the AgNO_3_ dosage. The silver load proved to be important also for the properties of the AgNP–rGO hybrid synthesized in the presence of poly vinyl pyrrolidone (Naqvi et al., 2019). Another type of hybrid material was obtained by the photochemical reduction of AgNO_3_ with graphene oxide nanocolloid (GON) spherical silver cores stabilized by the reduced GON, which serves as both reducing and stabilizing agent (Kim et al., 2017). The Ag@GON nanoparticles showed great stability. Cao et al. (2020) designed an effective SERS support using silver nanoparticles embedded in multilayer graphene. The highest enhancement was reached for the cross section of the membrane composed of the water-dispersible graphene and silver nanoparticles. Authors attributed the high enhancement to the plasmonic coupling between the silver nanoparticles resulting from the optimized distance between silver nanoparticles.

**FIGURE 1:**
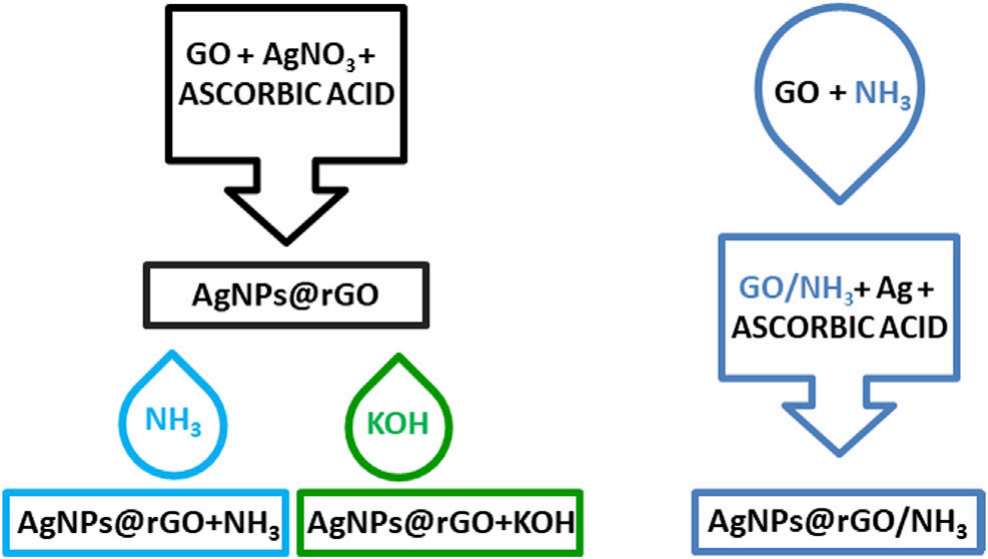
Overview of studied composites.

The listed examples indicate that the role of GO in SERS enhancement is still a subject of discussion and there are many possible factors which influence the SERS properties of GO itself or the properties of hybrid materials involving GO. Recently, we have shown that simple room temperature treatment of GO with ammonia solution improves the SERS enhancement factors for GO composites with gold nanoparticles (Kasztelan et al., 2021). In this contribution, we study silver composites synthesized by the simultaneous reduction of GO and silver ions using ascorbic acid as a mild reducing agent. The concentration of ascorbic acid was optimized to obtain the highest SERS enhancement. PATP was used as the SERS probe. The composite with an optimized concentration of ascorbic acid was further conditioned using ammonia or potassium hydroxide solution. For comparison, we synthesized the composite using pristine GO and GO conditioned in the aqueous ammonia solution. The overview of the studied composites is illustrated in Figure 1. We demonstrate the differences between the SERS characteristics of the studied materials.

## Materials and Methods

### Chemicals and Reagents

All reagents were available commercially and were used without further purification. Graphite powder (1–2 μm, synthetic), 4-aminothiophenol (4-ATP), potassium hydroxide (KOH), ascorbic acid (C_6_H_8_O_6_), and silver nitrate (AgNO_3_) were purchased from Sigma Aldrich and used without further purification. Sulfuric acid (H_2_SO_4_), potassium permanganate (KMnO_4_), and trisodium citrate (dihydrate, C_6_H_5_Na_3_O_7_ 2H_2_O) were purchased from Avantor Performance Materials Poland S.A. Hydrogen peroxide (H_2_O_2_, 30%) and ammonia solution (NH_4_OH, 25%) were purchased from Chempur, Poland. All solutions were prepared using distilled water (Millipore Milli-Q, 18.2 MΩ cm).

### Preparation of GO and Ammonia-Modified GO

Graphene oxide (GO) was synthesized using the modified Hummers–Offeman method. Typically, 6 g of graphite powder was mixed with 150 ml of concentrated sulfuric acid while being cooled in an ice bath to avoid overheating. Subsequently, 21 g of potassium permanganate was slowly added while stirring (maintaining the temperature below 30°C). After that, the mixture was stirred for 2 h, followed by slowly adding 150 ml of distilled water and 35 ml of 30% hydrogen peroxide. As-prepared GO solution was centrifuged at 6,000 rpm for 30 min. The supernatant was removed, and a portion of distilled water was added to the GO precipitate. This cleaning procedure was repeated 4 times. After that, the GO was left to dry.

To prepare ammonia-modified GO (GO/NH_3_), 25 mg of synthesized GO was mixed with 3.75 ml of 3% ammonia solution. After that, the solution was sonicated for 30 min and mixed using a magnetic stirrer for 24 h to allow the modification. Subsequently, GO/NH_3_ was centrifuged at 6,000 rpm for 30 min and left to dry at room temperature.

### Synthesis of AgNPs@rGO Composites

25 mg of GO was mixed with 25 ml of water and sonicated for 10 min. After that, 5 ml of GO solution was diluted using 95 ml of distilled water and sonicated for 30 min, followed by the addition of 3 ml of 0.04 M silver nitrate solution and 15 ml of 1% trisodium citrate solution. The mixture was further sonicated for 20 min. Subsequently, 2.25 ml of ascorbic acid was added to the mixture and stirred for 30 min using a magnetic stirrer. Three different composites were synthesized using different concentrations of ascorbic acid (0.002, 0.01, and 0.05 M) in order to evaluate the optimal concentration of the reducing agent. The same approach was used to synthesize the AgNPs@rGO/NH_3_ composite, where GO was replaced with GO/NH_3_. In this case, 0.01 M solution of ascorbic acid was used.

### Preparation of SERS Substrates

AgNPs@rGO and AgNPs@rGO/NH_3_ substrates were prepared by immersing ITO glass for 24 h in diluted solutions of AgNPs@rGO or AgNPs@rGO/NH_3_, respectively. The concentration of AgNPs@rGO and AgNPs@rGO/NH_3_ was equal to 1 mg/ml. AgNPs@rGO + NH_3_ and AgNPs@rGO + KOH substrates were prepared by dropping 100 μl of 3% ammonia solution or 3% KOH solution, respectively, on the AgNPs@rGO substrate. For SERS measurements, ethanol solutions of 4-PATP with concentrations from 10^−7^ to 10^−3^ M were deposited onto substrates by simply dropping 20 µL of the solution on the SERS substrate.

### Infrared Spectroscopy

Infrared spectra were recorded using a Nicolet iS50 FT-IR spectrometer (Thermo Scientific) with a DTGS detector. For all experiments, an iTR-attenuated total reflection accessory with a diamond crystal was used. Samples were prepared by drop-casting a small amount of the diluted composite on the diamond crystal. All experiments were performed with a resolution of 4 cm^−1^, and typically, 32 scans were taken for each sample.

### Raman Spectroscopy

Raman spectra were recorded using a DXR Raman microscope (Thermo Scientific) with a 50×/0.50 NA objective. In all measurements, the exposure time was 1 s, and typically, 4 scans were collected. To ensure repeatability, 10 spectra were collected in random spots on the sample and averaged for each concentration of 4-PATP. For each experiment, green laser (532 nm) was used as a source of excitation.

### UV–Vis Spectroscopy

UV–Vis spectra were recorded using a UV–Vis Lambda 650 spectrophotometer (PerkinElmer) with a variable slit. The spectral resolution was equal to 0.2 nm.

Spectra of the AgNPs@rGO composites synthesized using different concentrations of ascorbic acid were collected as diluted solutions in a quartz cuvette with an optical path length of 10 mm. Spectra of the composites treated with NH_3_ and KOH solutions were collected as thin layers deposited on ITO glass prepared in the same way as the SERS platforms.

### Scanning Electron Microscopy

The morphology of the SERS composites was investigated using a Merlin field emission scanning electron microscope system (Zeiss, Germany) at an operating voltage of 3 kV. Samples were prepared in a manner identical to that used for the SERS substrates.

## Results and Discussion

### Influence of the Ascorbic Acid Concentration on AgNPs@rGO Composites

**FIGURE 2:**
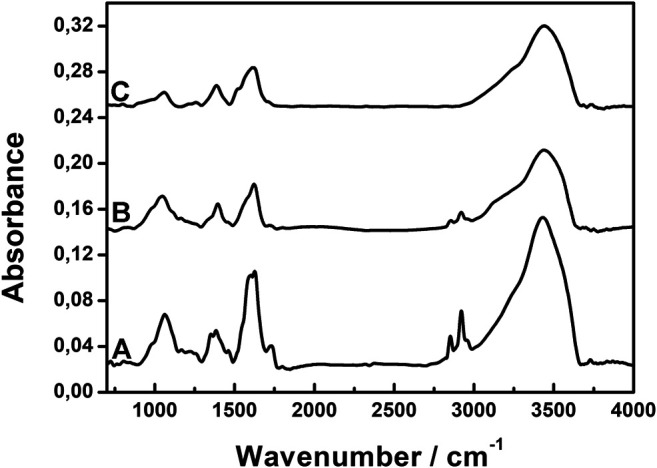
Infrared spectra of AgNPs@rGO composites synthesized using various concentrations of ascorbic acid: 0.002 M **(A)**; 0.01 M **(B)**; and 0.05 M **(C)**.

To optimize the concentration of the reagents for the synthesis of AgNPs@rGO, the following concentrations of the ascorbic acid solution were used: 0.002, 0.01, and 0.05 M. The infrared spectra of the obtained products were compared to the spectrum of the pristine GO. Figure 2 shows the typical spectra of all the AgNPs@rGO synthesized composites.

All spectra of the composites show no clear band due to the epoxide groups, which typically occur at 1,225 cm^−1^ in the GO spectrum (Świetlikowska et al., 2013), suggesting that the opening of the epoxide ring occurs easily—already at a low concentration of the reducing agent. In all composites, GO is at least partially reduced; therefore, we label them “AgNPs@rGO”. The increasing concentration of the ascorbic acid generally causes the diminishing of band intensities. The band at 1,730 cm^−1^, corresponding to the COOH groups, disappears, suggesting the removal of the COOH groups. But, the absence of the 1,730cm^−1^ band can also be attributed to the dissociation of the carboxylic groups. The antisymmetric and symmetric bands of COO^−^ are expected at 1,620 and 1,370 cm^−1^ (Socrates, 2001). There are bands at close frequencies observed in the spectra of AgNPs@rGO, but they can come from the citric ligands attached to silver nanoparticles. Infrared spectra cannot discern between the carboxylic groups coming from GO and the citric ligands attached to silver nanoparticles. Probably, both types of carboxylic groups are present in the studied samples. Spectra of all AgNPs@rGO composites also show band characteristics for OH surface groups. The OH stretching mode gives rise to the broad band with the maximum at 3,440 cm^−1^. The band at 1,060 cm^−1^ probably corresponds to the C–O stretching mode of the C–OH groups.

**FIGURE 3:**
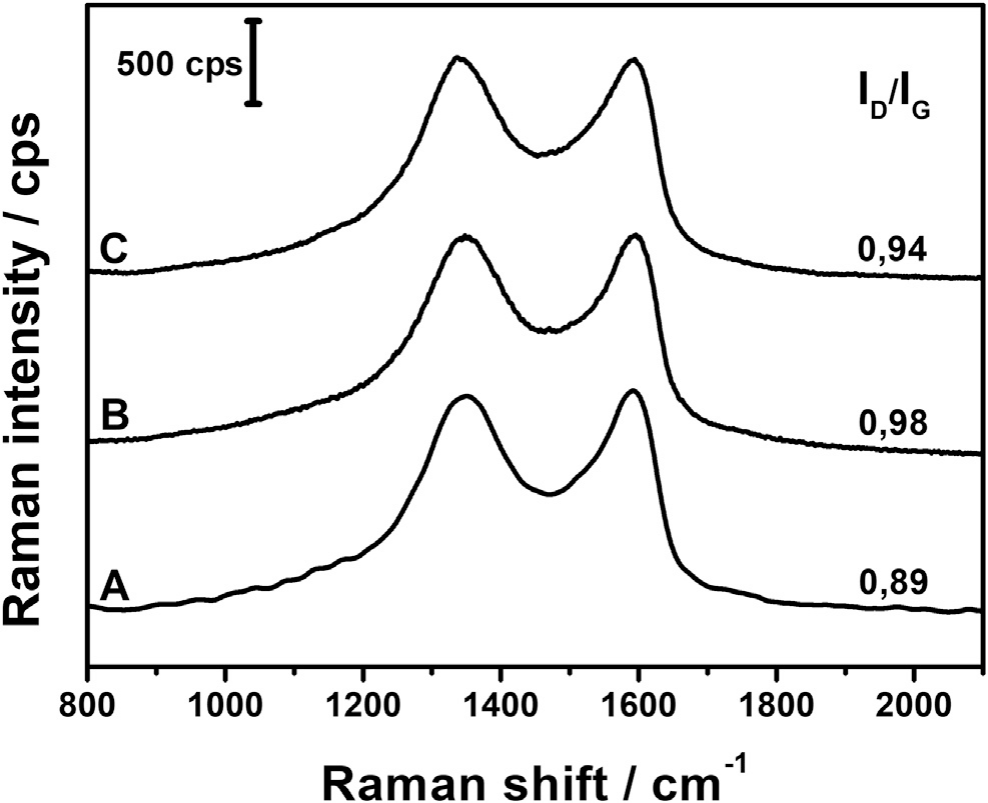
Raman spectra of AgNPs@rGO composites synthesized using various concentrations of ascorbic acid: 0.002 M **(A)**; 0.01 M **(B)**; and 0.05 M **(C)**.

Raman spectra of the composites were collected and are presented in Figure 3. In the typical spectrum, two main bands are observed in the fingerprint region. The band around 1,590 cm^−1^, called the G band, is due to the doubly degenerated C–C stretching mode of sp^2^ carbons, while the band around 1,340 cm^−1^ originates from sp^3^ defects and disruptions in the graphene layers (Beams et al., 2015). The spectra of all three composites are very similar, with only slight shifts in the band positions. The I_D_/I_G_ ratio can be utilized to evaluate the relative amount of defects in the GO plane since it increases with the increase in the I_D_/I_G_ ratio. Figure 3 shows that there is no significant change between the ratios in all three composites, which indicates that the different concentrations of ascorbic acid used in the syntheses have only a minute impact on the carbon structure of GO layers.

**FIGURE 4:**
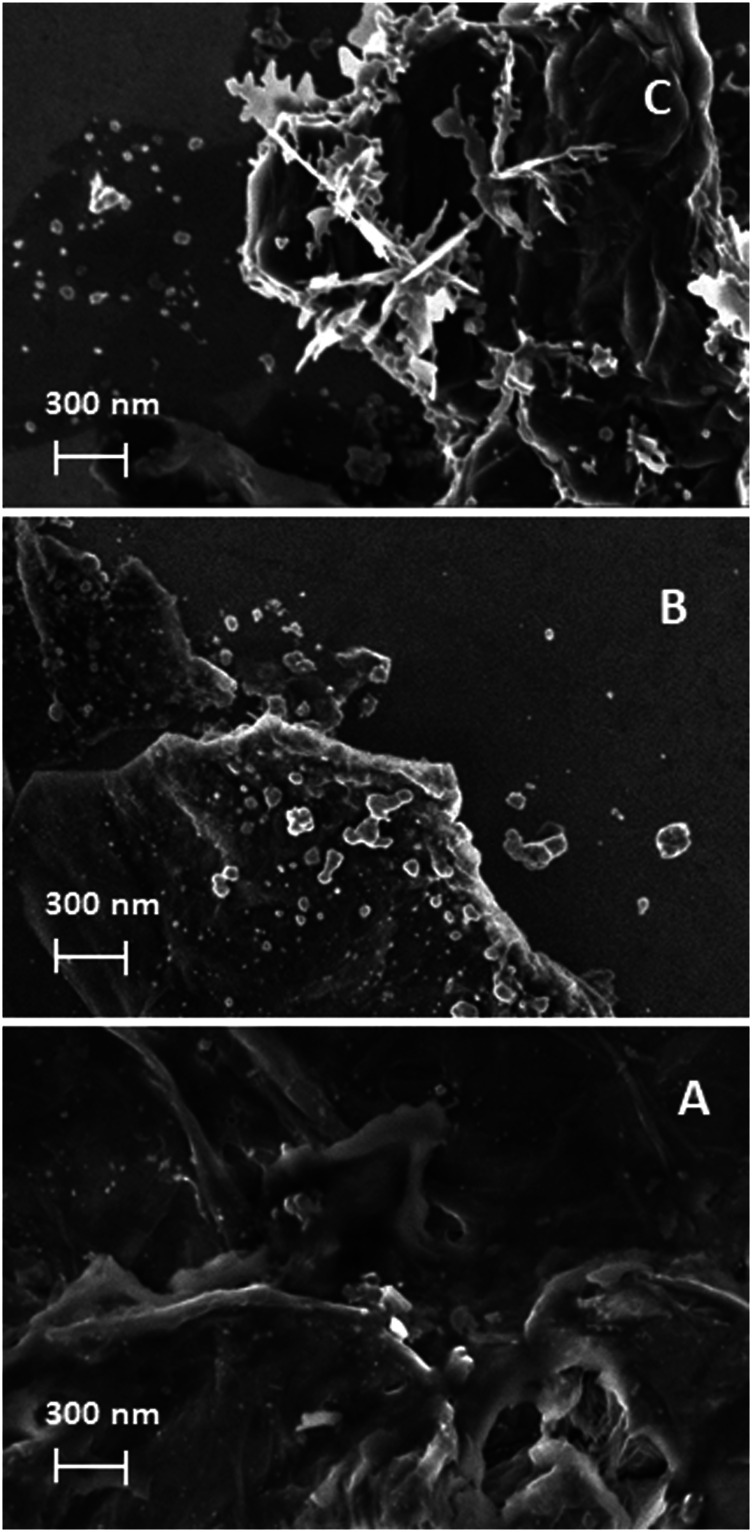
SEM images of AgNPs@rGO composites synthesized using various concentrations of ascorbic acid: 0.002 M **(A)**; 0.01 M **(B)**; and 0.05 M **(C)**.

The example SEM pictures of the composites are shown in Figure 4. The composite obtained at the concentration of ascorbic acid equal to 0.002 M shows isolated spherical silver structures stuck to the GO planes. A higher concentration of the reducing agent yields a composite with numerous spherical silver structures. A further increase in the concentration of the reducing agent causes the formation of irregular silver structures.

**FIGURE 5:**
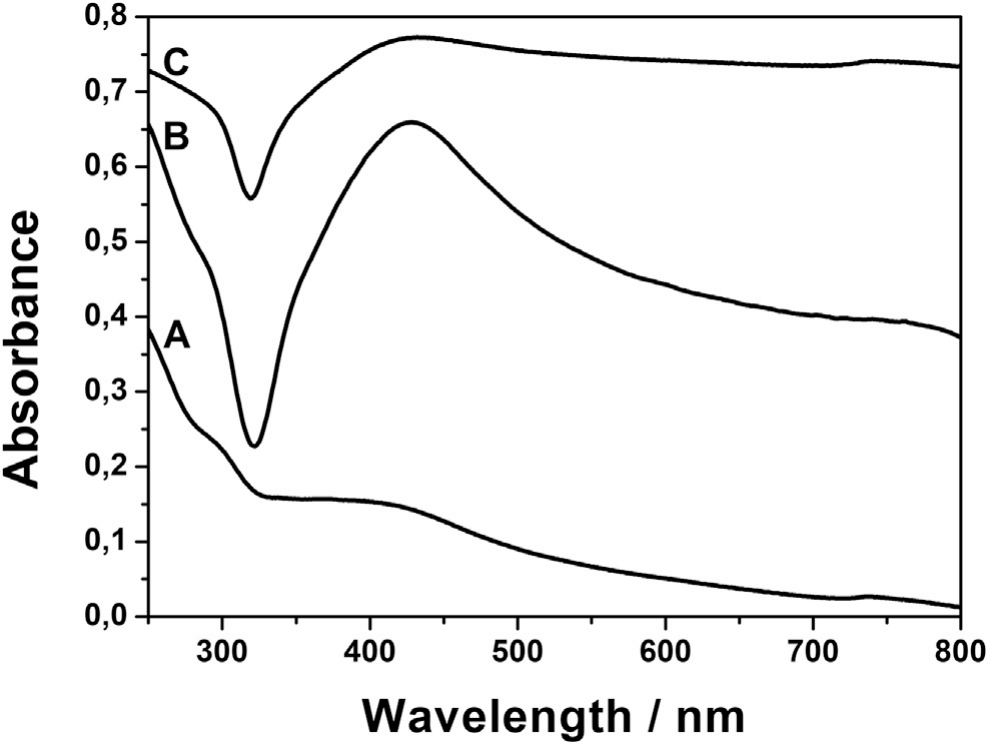
UV–Vis spectra of AgNPs@rGO composites synthesized using various concentrations of ascorbic acid: 0.002 M **(A)**; 0.01 M **(B)**; and 0.05 M **(C)**.

The UV–Vis spectra of the composites (Figure 5) show plasmonic absorption of silver at ca. 420 nm. The intensity of the plasmon bands is strongest for the intermediate concentration of the reducing agent. The composite obtained at the highest concentration of ascorbic acid shows a rather broad absorption without the clear maximum, which is related to the nonhomogeneous distribution of the sizes and shapes of the silver structures obtained under these conditions.

**FIGURE 6:**
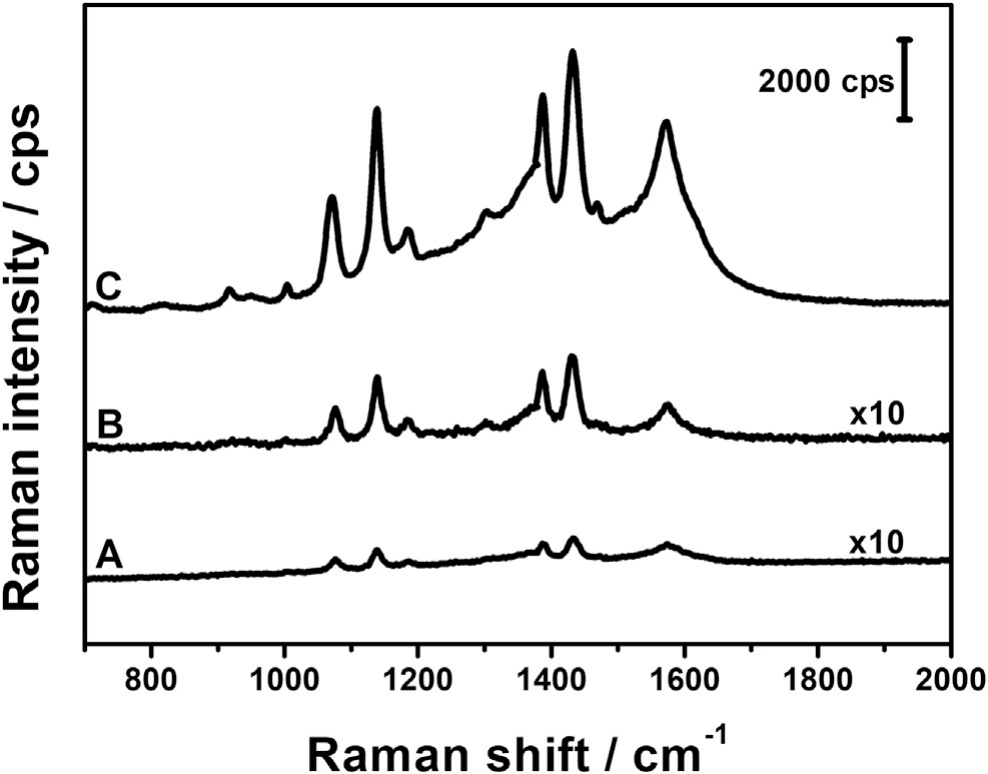
SERS spectra of PATP (10^−4^ M) adsorbed on AgNPs@rGO composites synthesized using various concentrations of ascorbic acid: 0.002 M **(A)**; 0.05 M **(B)**; and 0.01 M **(C)**.

The obtained composites were examined as possible SERS platforms. Figure 6 compares the typical spectra of PATP adsorbed on the three AgNPs@rGO composites. The spectra were recorded by drop-casting 20 µl of the 10^−4^ M solution of PATP on the studied support. There are five bands at 1,074, 1,136, 1,386, 1,430, and 1,573 cm^−1^, which are repeated in all the spectra in Figure 6. These bands are characteristic for p,p′-dimercaptoazobenzene (DMAB). It has been reported before that PATP undergoes a photochemical reaction on the silver supports, producing DMAB (Huang et al., 2010) and graphene-coated Ag bowtie nano-antenna arrays (Dai et al., 2015). The occurrence of DMAB bands indicates that AgNPs@rGO composites catalyze the photochemical dimerization of PATP.

Comparing the intensities of the SERS spectra shown in Figure 6, the highest is observed for the composite obtained at the intermediate concentration of the reagent. The highest SERS intensity correlates with the highest intensity of plasmonic absorption in the UV–Vis spectra. The synthesis with the intermediate concentration of ascorbic acid has been chosen for further studies.

### Influence of the Treatment of AgNPs@rGO With Ammonia and KOH Solutions

#### Structure and Morphology

**FIGURE 7:**
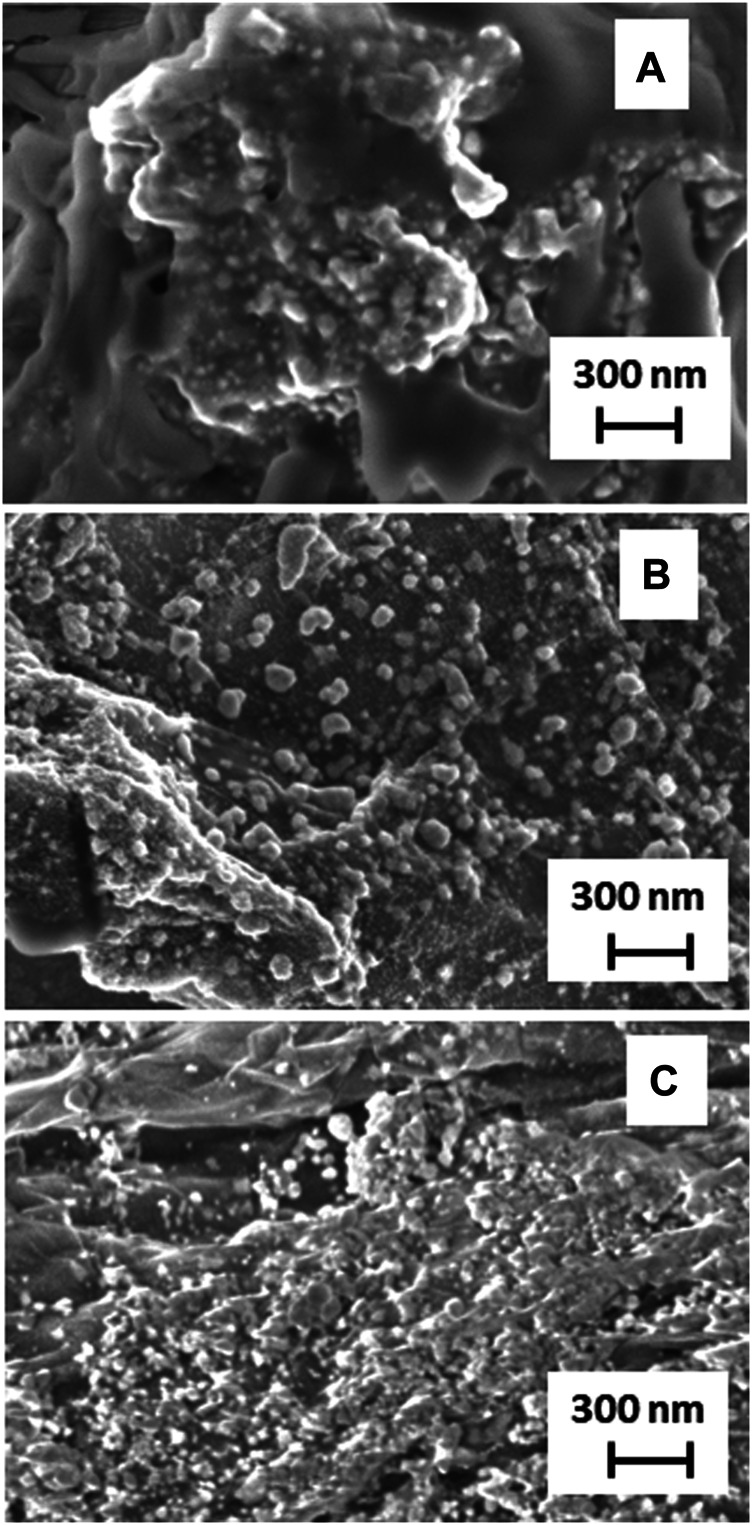
SEM images of AgNPs@rGO/NH3 **(A)**; AgNPs@rGO + NH_3_ **(B)**; and AgNPs@rGO + KOH **(C)**.

The AgNPs@rGO composite has been treated by a simple drop-casting of the ammonia solution or the KOH solution. The treated samples are labeled AgNPs@rGO + NH_3_ and AgNPs@rGO + KOH, respectively. For comparison, we have also synthesized a composite using the ammonia-treated GO, which is labeled AgNPs@rGO/NH_3_. Such treatment causes the partial reduction of GO, as shown by previous studies (Liu et al., 2015; Park et al., 2017). The morphology of the obtained samples has been studied by SEM. Figure 7 presents typical images. Comparing the images of AgNPs@rGO (Figure 4B) and AgNPs@rGO/NH_3_ (Figure 7A), it could be noticed that the composite synthesized using the ammonia-treated GO contains more densely packed silver nanostructures having a similar size to those of the one synthesized with pristine GO. Such an observation can be rationalized by the easier adsorption of silver ions on the ammonia-treated GO, leading to the formation of a more densely packed layer of silver nanostructures.

The casting of the ammonia solution after the synthesis causes less clear differences, as can be seen when comparing pictures of AgNPs@rGO (Figure 4B) and AgNPs@rGO + NH_3_ (Figure 7B). On the contrary, the drop-casting of the KOH solution on AgNPs@rGO causes a change of the morphology. The rGO planes seem more flexible, and part of them covers the silver nanoparticles in a loose manner.

**FIGURE 8:**
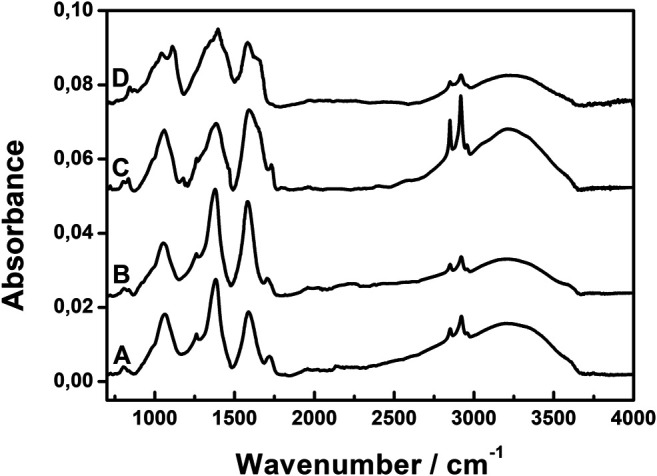
Infrared spectra of AgNPs@rGO **(A)**; AgNPs@rGO/NH_3_ **(B)**; AgNPs@rGO + NH_3_ **(C)**; and AgNPs@rGO + KOH **(D)**.

To get more information about the structure on the molecular level, we studied the infrared spectra of the composites. Figure 8 shows the typical spectra of the composites. The AgNPs@rGO and AgNPs@rGO/NH_3_ show very similar spectra, indicating that the conditioning of GO in ammonia before the synthesis of the composite has a negligible effect on the surface composition of GO in the final product.

On the contrary, adding the ammonia solution after the synthesis implies changes in the infrared spectra. The bands at 1,060, 1,380, and 1,585 cm^−1^ become noticeably broader. These bands involve contributions from the C–OH and COO^−^ groups; therefore, changes of their bandwidth suggest that the ammonia solution changes the interaction between the oxygen functional groups and the silver nanoparticles. The diminishing of the band due to COOH implies an increase of the dissociated COO^−^ groups, and the counter ions are possibly NH_4_^+^ ions. The characteristic bands of NH_4_^+^ typically occur at 1,400 cm^−1^—the N–H bending mode—and around 3,300 cm^−1^—the N–H stretching mode. Due to the strong bands of COO^−^ and OH groups, these bands are difficult to discern.

Dropping the KOH solution on AgNPs@rGO causes even more noticeable changes in the infrared spectra. The band at 1,730 cm^−1^ due to COOH disappears, indicating complete dissociation of the COOH surface groups. The bands at 1,060, 1,380, and 1,585 cm^−1^ are very broad like in the case of the ammonia-treated composite. Unlike in the case of ammonia, the band near 1,600 cm^−1^ shows two components, suggesting that there might be two types of COO^−^, which have different molecular surroundings, for example, interacting with silver atoms and not interacting. The band at 1,060 cm^−1^ gets a new component as well, which is located at 1,110 cm^−1^, suggesting that the surroundings of C–OH groups also change upon the addition of KOH.

**FIGURE 9:**
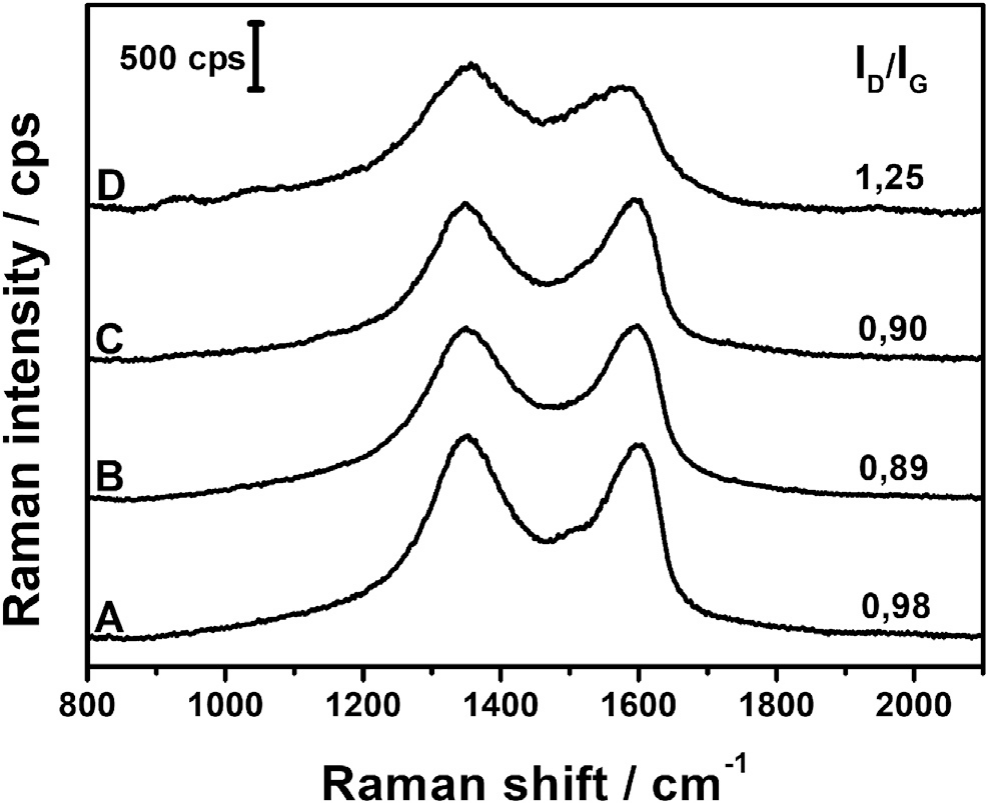
Raman spectra of AgNPs@rGO **(A)**; AgNPs@rGO/NH_3_ **(B)**; AgNPs@rGO + NH_3_ **(C)**; and AgNPs@rGO + KOH **(D)**.

Raman spectra of AgNPs@rGO composites treated with basic solutions are shown in Figure 9. In case of the AgNPs@rGO, AgNPs@rGO/NH_3_, and AgNPs@rGO + NH_3_ composites, there are only minor shifts in the G and D band positions, and the I_D_/I_G_ ratios do not differ much from each other, although for composites treated with ammonia, the slightly lower ratio values may indicate a partial recovery of sp^2^ hybridized carbon lattice. Major changes occur after treatment with KOH solution. The I_D_/I_G_ ratio increases from 0.98 to 1.25, indicating the formation of new defects after the treatment with KOH. A significant decrease of the G band position toward the lower Raman shift (1,577 cm^−1^ compared to 1,598 cm^−1^ for the pristine composite) and the overall broadening of both G and D bands can be caused by the considerable influence of KOH on the oxygen content in the composite (Kudin et al., 2008; Claramunt et al., 2015). Red-shift of the G band is usually observed along with diminishing content of oxygen surface groups, while the broadening of bands might be the result of the formation of new oxygen species that give a contribution to the general widening of the bands as indicated by infrared spectra.

#### Plasmonic Absorption and SERS Enhancement

**FIGURE 10:**
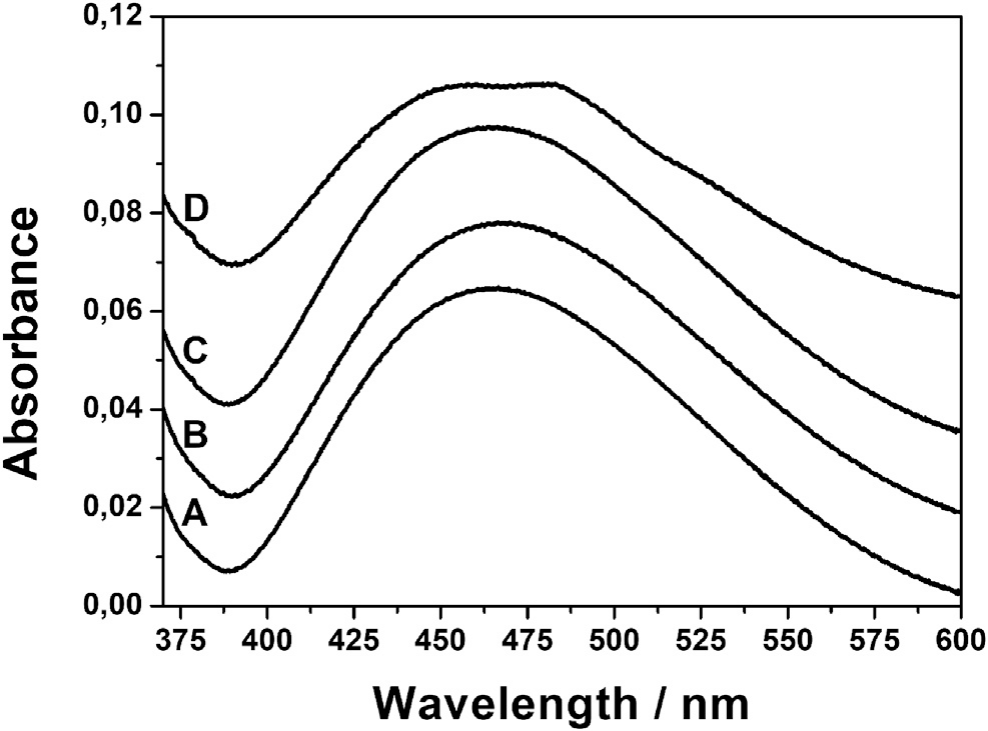
UV–Vis spectra of AgNPs@rGO **(A)**; AgNPs@rGO + NH_3_ **(B)**; AgNPs@rGO/NH_3_ **(C)**; and AgNPs@rGO + KOH **(D)**.

To verify whether the structural changes observed in the infrared spectra influence the plasmonic properties of the composites, the UV–Vis absorption spectra were studied. Figure 10 shows the typical spectra in the range of silver plasmonic absorption. The spectra of AgNPs@rGO, AgNPs@rGO + NH_3_, and AgNPs@rGO/NH_3_ show only minute differences in the position of the maximum of absorption, being, respectively, 464, 470, and 462 nm. The plasmonic band of AgNPs@rGO + KOH is split into two components at 458 and 485 nm. The plasmon band is sensitive to the changes in the vicinity of nanoparticles; therefore, the slight shifts and the split of the plasmonic band suggest changes in the interaction between silver nanoparticles and rGO. The UV–Vis results correlate well with the infrared spectra.

**FIGURE 11:**
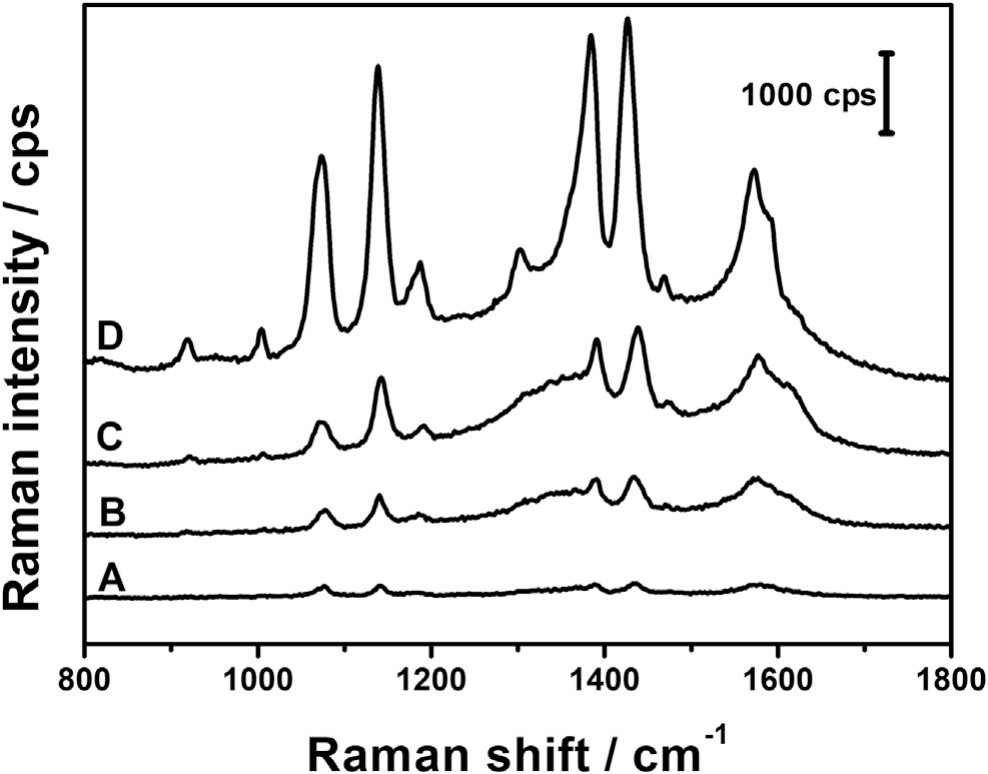
SERS spectra of PATP (10^−6^M) adsorbed on AgNPs@rGO **(A)**; AgNPs@rGO + NH_3_ **(B)**; AgNPs@rGO/NH_3_ **(C)**; and AgNPs@rGO + KOH **(D)**.

The SERS properties of the composites treated with ammonia or KOH were tested using PATP as a probe. Figure 11 compares SERS spectra obtained at the concentration of PATP equal to10^−6^ M. For all supports, the spectra show bands typical for DMAB, so all composites catalyze the transformation from PATP to DMAB. The SERS efficiency of the supports can be ordered as follows: AgNPs@rGO, AgNPs@rGO + NH_3_, AgNPs@rGO/NH_3_, and AgNPs@rGO + KOH. The high enhancement observed for the composite treated with KOH correlates with the shift of the plasmon absorption band.

The differences between the three other composites probably result from the adsorption of ammonium ions on GO, which contributes to the SERS enhancement by the dipole contribution (Wang et al., 2020).

#### Summary and Discussion of the NH_3_ and KOH Influence on SERS

The SERS efficiency of the studied hybrid materials can be placed in an order: AgNPs@rGO < AgNPs@rGO + NH_3_ < AgNPs@rGO/NH_3_ < AgNPs@rGO + KOH.

The differences can be rationalized based on infrared, Raman, UV/VIS, and SEM characteristics of the studied materials. The treatment of AgNPs@rGO with ammonia after the synthesis (producing AgNPs@rGO + NH_3_) does influence the infrared spectra, causing the diminishing of the COOH bands and the broadening of the bands due to the COO^−^ and C–OH groups. More negatively charged COO^−^ groups imply the probable bonding of NH_4_^+^ ions as counter ions. The bands of NH_4_^+^ ions are visible in the infrared spectrum of AgNPs@rGO + NH_3_. The other techniques (Raman, UV/VIS, and SEM) do not show significant differences between AgNPs@rGO and AgNPs@rGO + NH_3_. The differences between these two materials are thus only the presence of COO^−^ and NH_4_^+^ ions. We suppose that ions may contribute to the SERS enhancement via the dipole mechanism (Wang et al., 2020). Such enhancement could explain why AgNPs@rGO + NH_3_ enhances the SERS spectra better than AgNPs@rGO.

Unlike AgNPs@rGO + NH_3_, AgNPs@rGO/NH_3_ shows similar infrared spectrum to AgNPs@rGO (Figures 8A,B). Such similarity suggests that the molecular structure of the two composites is the same, and it is not responsible for different SERS properties. The difference is visible in SEM images. The AgNPs@rGO/NH_3_ contains more densely packed silver structures than AgNPs@rGO. The silver particles have similar sizes and shapes. The similar size and shape of the silver particles is consistent with the similarity of the UV–Vis spectra (Figures 10A,B). The higher load of AgNPs makes the average distance between the silver particles smaller, which contributes to the stronger coupling between them (Nam et al., 2016).

The potassium hydroxide exerts the biggest influence on AgNPs@rGO. The changes are visible in infrared, Raman, and UV–Vis spectra and SEM images. The COOH band visible in the AgNPs@rGO infrared spectrum disappears in the AgNPs@rGO + KOH spectrum, indicating the dissociation of carboxylic groups. The potassium ions are probably attached to the COO^−^ groups for charge compensation. Ionic species on the surface contribute to the SERS enhancements via the dipole mechanism like in the case of AgNPs@rGO + NH_3_. Changes visible in the UV–Vis spectra also suggest that the electromagnetic part of the enhancement is changed. The band becomes asymmetric with a discernible new component. The increased UV–Vis absorption at the longer wavelength side probably contributes to the increased SERS enhancement because the absorption fits better to the excitation laser line.

## Conclusion

The concentration of ascorbic acid used for the synthesis of AgNPs@rGO nanohybrids has a significant impact on the obtained composite, including the shape and the amount of silver nanostructures formed on the GO layers. Simple treatment of the AgNPs@rGO composite with ammonia or potassium hydroxide solution causes changes in the shape of the plasmonic absorption band in the UV–Vis range and their SERS properties. The SERS efficiency of the studied hybrid materials can be placed in an order: AgNPs@rGO < AgNPs@rGO + NH_3_ < AgNPs@rGO/NH_3_ < AgNPs@rGO + KOH.

## Data Availability

The raw data supporting the conclusions of this article will be made available by the authors, without undue reservation.

## References

[B1] Beams R. Gustavo Cançado L. Novotny L. (2015). Raman Characterization of Defects and Dopants in Graphene. J. Phys. Condens. Matter 27, 083002. 10.1088/0953-8984/27/8/083002 25634863

[B2] Berbeć S. Żołądek S. Kulesza P. J. Pałys B. (2019). Silver Nanoparticles Stabilized by Polyoxotungstates. Influence of the Silver – Polyoxotungstate Molar Ratio on UV/Vis Spectra and SERS Characteristics. J. Electroanalytical Chem. 854, 113537. 10.1016/j.jelechem.2019.113537

[B3] Cao Z. He P. Huang T. Yang S. Han S. Wang X. (2020). Plasmonic Coupling of AgNPs Near Graphene Edges: A Cross-Section Strategy for High-Performance SERS Sensing. Chem. Mater. 32, 3813–3822. 10.1021/acs.chemmater.9b05293

[B4] Claramunt S. Varea A. López-Díaz D. Velázquez M. M. Cornet A. Cirera A. (2015). The Importance of Interbands on the Interpretation of the Raman Spectrum of Graphene Oxide, J. Phys. Chem. C, 119, 10123–10129. 10.1021/acs.jpcc.5b01590

[B5] Dai Z.-g. Xiao X.-h. Wu W. Zhang Y.-p. Liao L. Guo S.-s. (2015). Plasmon-driven Reaction Controlled by the Number of Graphene Layers and Localized Surface Plasmon Distribution during Optical Excitation. Light Sci. Appl. 4, e342. 10.1038/lsa.2015.115

[B6] Darrigues E. Dantuluri V. Nima Z. A. Vang-Dings K. B. Griffin R. J. Biris A. R. (2017). Raman Spectroscopy Using Plasmonic and Carbon-Based Nanoparticles for Cancer Detection, Diagnosis, and Treatment Guidance. Part 2: Treatment. Drug Metab. Rev. 49, 253–283. 10.1080/03602532.2017.1307387 28298144

[B7] Dina N. E. Raluca Gherman A. M. Colniță A. Marconi D. Sârbu C. (2021). Fuzzy Characterization and Classification of Bacteria Species Detected at Single-Cell Level by Surface-Enhanced Raman Scattering. Spectrochim Acta A. Mol. Biomol. Spectrosc. 247, 119149. 10.1016/j.saa.2020.119149 33188974

[B8] Fan W. Lee Y. H. Pedireddy S. Zhang Q. Liu T. Ling X. Y. (2014). Graphene Oxide and Shape-Controlled Silver Nanoparticle Hybrids for Ultrasensitive Single-Particle Surface-Enhanced Raman Scattering (SERS) Sensing. Nanoscale 6, 4843–4851. 10.1039/C3NR06316J 24664184

[B9] Huang Y. Fang Y. Yang Z. Sun M. (2010). Can P,p′-Dimercaptoazobisbenzene Be Produced from P-Aminothiophenol by Surface Photochemistry Reaction in the Junctions of a Ag Nanoparticle−Molecule−Ag (Or Au) Film?. J. Phys. Chem. C 114, 18263–18269. 10.1021/jp107305z

[B10] Kasztelan M. Słoniewska A. Gorzkowski M. Lewera A. Pałys B. Zoladek S. (2021). Ammonia Modified Graphene Oxide—Gold Nanoparticles Composite as a Substrate for Surface Enhanced Raman Spectroscopy. Appl. Surf. Sci. 554, 149060. 10.1016/j.apsusc.2021.149060

[B11] Kavitha C. (2018). Reduced Graphene Oxide/Nanoparticle Hybrid Structures: A New Generation Smart Materials for Optical Sensors. Mater. Today 10, 113. 10.1016/j.matpr.2018.01.040

[B12] Kim Y.-K. Kim S. Cho S.-P. Jang H. Huh H. Hong B. H. (2017). Facile One-Pot Photosynthesis of Stable Ag@graphene Oxide Nanocolloid Core@shell Nanoparticles with Sustainable Localized Surface Plasmon Resonance Properties. J. Mater. Chem. C 5, 10016–10022. 10.1039/C7TC03379F

[B13] Kudin K. N. Ozbas B. Schniepp H. C. Prud'homme R. K. Aksay I. A. Car R. (2008). Raman Spectra of Graphite Oxide and Functionalized Graphene Sheets. Nano Lett. 8, 36–41. 10.1021/nl071822y 18154315

[B14] Langer J. Jimenez de Aberasturi D. Aizpurua J. Alvarez-Puebla R. A. Auguié B. Baumberg J. J. (2020). Present and Future of Surface-Enhanced Raman Scattering. ACS Nano 14, 28–117. 10.1021/acsnano.9b04224 31478375 PMC6990571

[B15] Li A. Tang L. Song D. Song S. Ma W. Xu L. (2016). A SERS-Active Sensor Based on Heterogeneous Gold Nanostar Core-Silver Nanoparticle Satellite Assemblies for Ultrasensitive Detection of aflatoxinB1. Nanoscale 8, 1873–1878. 10.1039/C5NR08372A 26732202

[B16] Li Y. Zhao X. Zhang P. Ning J. Li J. Su Z. (2015). A Facile Fabrication of Large-Scale Reduced Graphene Oxide–Silver Nanoparticle Hybrid Film as a Highly Active Surface-Enhanced Raman Scattering Substrate. J. Mater. Chem. C 3, 4126–4133. 10.1039/C5TC00196J

[B17] Lin D. Qin T. Wang Y. Sun X. Chen L. (2014). Graphene Oxide Wrapped SERS Tags: Multifunctional Platforms toward Optical Labeling, Photothermal Ablation of Bacteria, and the Monitoring of Killing Effect. ACS Appl. Mater. Inter. 6, 1320–1329. 10.1021/am405396k 24380413

[B18] Liu C. He C. Xie T. Yang J. (2015). Reduction of Graphite Oxide Using Ammonia Solution and Detection Cr(VI) with Graphene-Modified Electrode. Fullerenes, Nanotubes and Carbon Nanostructures 23, 125–130. 10.1080/1536383X.2013.833914

[B19] Lombardi J. R. (2017). The Theory of Surface-Enhanced Raman Scattering on Semiconductor Nanoparticles; toward the Optimization of SERS Sensors. Faraday Discuss. 205, 105–120. 10.1039/C7FD00138J 28885632

[B20] Luo S.-C. Sivashanmugan K. Liao J.-D. Yao C.-K. Peng H.-C. (2014). Nanofabricated SERS-Active Substrates for Single-Molecule to Virus Detection *In Vitro*: A Review. Biosens. Bioelectron. 61, 232–240. 10.1016/j.bios.2014.05.013 24892785

[B21] Maddali H. Miles C. E. Kohn J. O'Carroll D. M., (2021). Optical Biosensors for Virus Detection: Prospects for SARS‐CoV‐2/COVID‐19, ChemBioChem, 22, 1176, 1189. 10.1002/cbic.202000744 33119960 PMC8048644

[B22] Mohammadi A. Nicholls D. Docoslis A. (2018). Improving the Surface-Enhanced Raman Scattering Performance of Silver Nanodendritic Substrates with Sprayed-On Graphene-Based Coatings. Sensors 18, 3404. 10.3390/s18103404 PMC6209902 30314312

[B23] Nam J. M. Oh J. W. Lee H. Suh Y. D. (2016). Plasmonic Nanogap-Enhanced Raman Scattering with Nanoparticles. Acc. Chem. Res. 49, 2746–2755. 10.1021/acs.accounts.6b00409 27993009

[B24] Naqvi T. K. Srivastava A. K. Kulkarni M. M. Siddiqui A. M. Dwivedi P. K. (2019). Silver Nanoparticles Decorated Reduced Graphene Oxide (rGO) SERS Sensor for Multiple Analytes. Appl. Surf. Sci. 478, 887–895. 10.1016/j.apsusc.2019.02.026

[B25] Otto A. (2005). The 'chemical' (Electronic) Contribution to Surface-Enhanced Raman Scattering. J. Raman Spectrosc. 36, 497–509. 10.1002/jrs.1355

[B26] Park M.-S. Lee S. Lee Y.-S. (2017). Mechanical Properties of Epoxy Composites Reinforced with Ammonia-Treated Graphene Oxides. Carbon Lett. 21, 1–7. 10.5714/CL.2017.21.001

[B27] Socrates G. (2001). Infrared and Raman Characteristic Group Frequencies. 3rd ed. Chichester: John Wiley & Sons.

[B28] Świetlikowska A. Gniadek M. Pałys B. (2013). Electrodeposited Graphene Nano-Stacks for Biosensor Applications. Surface Groups as Redox Mediators for Laccase. Electrochimica Acta 98, 75–81. 10.1016/j.electacta.2013.03.055

[B29] Wang L. Zhang Y. Yang Y. Zhang J. (2018). Strong Dependence of Surface Enhanced Raman Scattering on Structure of Graphene Oxide Film. Materials 11, 1199. 10.3390/ma11071199 PMC6073250 30002326

[B30] Wang R.-C. Chen Y.-H. Huang H.-H. Lin K.-T. Jheng Y.-S. Liu C.-Y. (2020). Justification of Dipole Mechanism over Chemical Charge Transfer Mechanism for Dipole-Based SERS Platform with Excellent Chemical Sensing Performance. Appl. Surf. Sci. 521, 146426. 10.1016/j.apsusc.2020.146426

[B31] Wu P. Gao Y. Zhang H. Cai C. (2012). Aptamer-Guided Silver-Gold Bimetallic Nanostructures with Highly Active Surface-Enhanced Raman Scattering for Specific Detection and Near-Infrared Photothermal Therapy of Human Breast Cancer Cells. Anal. Chem. 84, 7692–7699. 10.1021/ac3015164 22925013

[B32] Yan T. Zhang L. Jiang T. Bai Z. Yu X. Dai P. (2017). Controllable SERS Performance for the Flexible Paper-like Films of Reduced Graphene Oxide. Appl. Surf. Sci. 419, 373–381. 10.1016/j.apsusc.2017.05.052

[B33] Yang H. Hu H. Ni Z. Poh C. K. Cong C. Lin J. (2013). Comparison of Surface-Enhanced Raman Scattering on Graphene Oxide, Reduced Graphene Oxide and Graphene Surfaces. Carbon 62, 422–429. 10.1016/j.carbon.2013.06.027

[B34] Yu X. Cai H. Zhang W. Li X. Pan N. Luo Y. (2011). Tuning Chemical Enhancement of SERS by Controlling the Chemical Reduction of Graphene Oxide Nanosheets. ACS Nano 5, 952–958. 10.1021/nn102291j 21210657

[B35] Zhang N. Tong L. Zhang J. (2016). Graphene-Based Enhanced Raman Scattering toward Analytical Applications. Chem. Mater. 28, 6426–6435. 10.1021/acs.chemmater.6b02925

[B36] Zhang Y. Zhao S. Zheng J. He L. (2017). Surface-enhanced Raman Spectroscopy (SERS) Combined Techniques for High-Performance Detection and Characterization. Trac Trends Anal. Chem. 90, 1–13. 10.1016/j.trac.2017.02.006

[B37] Zou S. Ma L. Li J. Liu Y. Zhao D. Zhang Z. (2019). Ag Nanorods-Based Surface-Enhanced Raman Scattering: Synthesis, Quantitative Analysis Strategies, and Applications. Front. Chem. 7, 376. 10.3389/fchem.2019.00376 31214564 PMC6558050

[B38] Zrimsek A. B. Chiang N. Mattei M. Zaleski S. McAnally M. O. Chapman C. T. (2017). Single-Molecule Chemistry with Surface- and Tip-Enhanced Raman Spectroscopy. Chem. Rev. 117, 7583–7613. 10.1021/acs.chemrev.6b00552 28610424

